# Plucked Human Hair Shafts and Biomolecular Medical Research

**DOI:** 10.1155/2013/620531

**Published:** 2013-10-31

**Authors:** Kevin Schembri, Christian Scerri, Duncan Ayers

**Affiliations:** ^1^Department of Physiology & Biochemistry, Faculty of Medicine and Surgery, University of Malta, Msida MSD2080, Malta; ^2^Department of Pathology, Faculty of Medicine and Surgery, University of Malta, Msida MSD2080, Malta; ^3^Manchester Institute of Biotechnology, Faculty of Medical and Human Sciences, The University of Manchester, Manchester M1 7DN, UK

## Abstract

The hair follicle is a skin integument at the boundary between an organism and its immediate environment. The biological role of the human hair follicle has lost some of its ancestral importance. However, an indepth investigation of this miniorgan reveals hidden complexity with huge research potential. An essential consideration when dealing with human research is the awareness of potential harm and thus the absolute need not to harm—a rule aptly qualified by the Latin term “primum non nocere” (first do no harm). The plucked hair shaft offers such advantages. The use of stem cells found in hair follicles cells is gaining momentum in the field of regenerative medicine. Furthermore, current diagnostic and clinical applications of plucked hair follicles include their use as autologous and/or three-dimensional epidermal equivalents, together with their utilization as surrogate tissue in pharmacokinetic and pharmacodynamics studies. Consequently, the use of noninvasive diagnostic procedures on hair follicle shafts, posing as a surrogate molecular model for internal organs in the individual patient for a spectrum of human disease conditions, can possibly become a reality in the near future.

## 1. Introduction

The hair follicle is a skin integument at the boundary between an organism and its immediate environment. It is the evolutionary relative of the scale, feather, and nail, integuments that have served an essential role in the survival of organisms [1]. The biological role of the human hair follicle has lost some of its ancestral importance; however, an indepth investigation of this miniorgan reveals hidden complexity with huge research potential. The authors Paus and Foitzik describe the hair follicle as having a unique mammalian characteristic with a stem cell-rich, prototypic neuroectodermal-mesodermal interaction system. It is described as a mammalian organ undergoing cyclic transformations from stages of rapid growth (anagen) to apoptosis-driven regression (catagen) and back to anagen, via an interspersed period of relative quiescence (telogen) that persists throughout the animal's lifetime [2]. 

This miniorgan has been studied both *in vitro* and *in vivo*, in animals and in humans. Every approach has advantages and disadvantages, but there is no doubt that human research, allowing minimal interference, produces highly relevant results. An essential consideration when dealing with human research is the awareness of potential harm, and thus the absolute need not to harm a rule aptly qualified by the Latin term “primum non nocere” (first do no harm). The plucked hair shaft offers such advantages.

Hair shafts are common, small, and easily obtainable without a major discomfort to the individual participating in the research. Hair shafts represent human tissue that can be sampled over different time points. This miniorgan has both neuroectodermal and mesodermal origins as well as acting as a source of stem cells. This paper will mainly focus on the use of the plucked hair shaft for human medical research and its emerging potential. 

## 2. Plucked Hair Shaft Anatomy and Integrity

**Figure 1:**
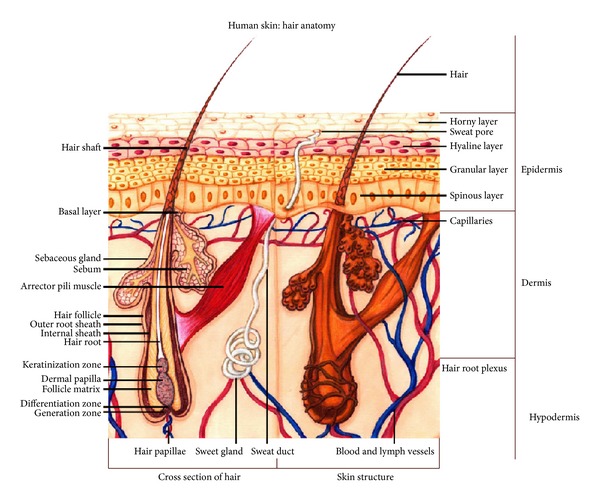
Graphic representation of a typical hair follicle, depicting the regions allowing for follicle generation and differentiation, together with the dermal papilla and follicle matrix.

The intact hair follicle (see Figure 1) can be simply described from a histological point of view as a minute cluster of uniform epithelial cells, adjacent to a similar sized aggregation of uniform mesenchymal cells. It is an organ composed of five or six concentric cylinders, each of which is composed of cells of a distinctive type, synthesizing their own distinctive set of proteins [3]. A detailed biology of the intact hair follicle can be found in the article published by Paus and Cotsarelis [4].

Obtaining an intact hair follicle is only possible by means of a skin biopsy. This invasive procedure restricts the availability, mainly to tissue obtained during other surgical procedures such as the skin excess obtained during face-lift surgery and samples obtained during hair transplant procedures. Plucking of hair shafts is an alternative, less invasive technique. There is, however, the question of how many cells come up with the uprooting of the follicle and which types of cells come off with such method. 

Although the plucked hair shaft is clearly inferior in cellular quantity and complexity to an intact hair follicle as obtained by a biopsy, it does carry sufficient cellular mass to permit detailed scientific investigations. Moll employed the plucked hair follicle to identify regions with the greatest growth potential in culture as well as analysing gene expression and protein analyses in the different segments of the plucked follicles [5].

When comparing haematoxylin and eosin stained hair follicles derived from skin biopsies and from plucked hairs using light microscopy, Gho and colleagues demonstrated that most of the epithelial structures from the hair follicle do remain attached to the plucked hair [6]. The maintenance of integrity of the outer root sheet after hair plucking is a possible outcome and has been documented by Limat and colleagues [7]. Plucking of hair follicles permits the investigation into pigment cells, an approach undertaken in the 1950s by Barnicot and colleagues who were among the first to study the plucked hair shaft under electron microscopy [8]. Human anagen scalp hair bulbs were examined by light microscopy to investigate the anatomical effects of mechanical plucking [9]. Interestingly, the study demonstrated that anagen hair bulbs tear off in reproducible patterns [9]. Apart from the “typical” break conically surrounding the dermal papilla, the authors also describe four additional break forms [9]:

1. rupture of the hair around the upper third of the papilla resulting in dysplastic anagen hairs of the trichogram,
2. rupture of the hair well above the dermal papilla resulting in “broken” anagen hairs,
3. total removal of the proximal follicle epithelium with removal of the dermal papilla resulting in so-called papilla hairs of the trichogram [9];
4. the other effect of plucking on the hair follicle is the alteration of the mesenchymal sheath, giving rise to hemorrhages and oedema increasing the volume of both the dermal papilla and the underlying “papilla cushion” of Pinkus.

The break types described by Bassukas and Horstein are possibly due either to inappropriate plucking techniques or due to the different subphases of the anagen stage [9]. 

A system for staging plucked hair shafts has been described by Camidge and colleagues in their paper about the use of plucked human hair as a tissue that can be utilized for assessing the pharmacodynamic endpoints during drug development studies [10]. Hairs were examined by light microscopy to evaluate nuclear staining in terms of its presence/absence, the site of staining, and the stage of hair [10]. Each hair with a visible bulb and root sheath was photographed and staged (0, 1, 2, or 3), according to a bespoke system based on the distance of the lower margin of the sheath from the base of the bulb [10]. 

Stage 0 was defined as sheath encompassing the bulb, stage 1 < 150 *μ*m, stage 2 = 150–699 *μ*m, and stage 3 > 700 *μ*m. Hairs noted to be without visible bulbs and sheaths that had not been excluded previously at the initial by-eye examination were discarded [10]. 

The studies described above have set the basis for a reproducible scientific classification of plucked hair follicles, given the possible diverse effect of plucking on the histology of the tissue under investigation. 

## 3. Stem Cells and Plucked Hair Shafts

The epidermis harbours two stem cell repositories, one found in the basal layer of interfollicular epidermis and the other in the hair follicle. The use of stem cells found in hair follicles cells is gaining momentum in the field of regenerative medicine. This has created the need to identify the exact site in the hair follicle and to design simple methods to access such cells such as from plucked hair follicles that are readily available.

Moll performed studies on the localization of colony-forming cells in human plucked hair shafts [11]. The investigators utilised anagen scalp plucked hairs to confirm the presence of an intact outer root sheet as well as the proliferative potential of the different keratinocytes [11]. In order to achieve localisation, five segments of the outer root sheath (ORS), B1, B2, B3-1, B3-2, and B4, were delineated by means of microdissection. It was found that colony-forming ability was mostly marked in the intermediate part (B2) and the lower half of the central part (B3-1) [11]. The longest *in vitro* life span was found in the fragment B3-2 and the shortest in the fragment B1 (bulb) [11]. Since the high colony-forming ability cells localized in the lower central parts of the ORS keratinocytes are usually removed by plucking, the authors comment that they may, therefore, not represent stem cells but rather cells important for hair growth during a single cycle [11]. Cells with long life spans were localized in central parts of the outer root sheath close to the bulge area, whereas cells with long life spans also included in plucked hair follicles could be an immediate progeny of stem cells that would be segregated in the bulge area [11].

Gho and colleagues investigated and confirmed the presence of stem cells in plucked anagen hair follicles from scalp occipital area [6]. This was achieved by testing for cytokeratin 19, a marker claimed by Michel et al. to be positive for stem cells; they indirectly localised these cells [12]. It was also argued that, since stem cells required protection against apoptotic hair cycle, investigating apoptosis-suppressing Bcl-2 protein together with the absence of the apoptosis-promoting Bax would be another reliable method by which the investigators could look for the presence of stem cells [6].

Another reliable method to identify keratinocyte stem cells makes use of the fact that these cells are normally slow-cycling, hence can be identified experimentally as the “label-retaining cells” (LRCs) [13, 14]. In this approach, one labels all the cells in the epithelium by a repeated or continuous supply of tritiated thymidine, followed by a long chase period during which the label is lost from all the cycling, transit amplifying (TA) cells, allowing only cells that cycle slowly (the stem cells) to retain the label [15]. Slow-cycling cells of the hair follicle were found by Taylor and colleagues to be exclusively confined to a previously ignored area called the bulge, with that part of the outer root sheath marking the lowest point of the upper, permanent portion of the follicle, as well as the attachment site of the arrector pili muscle.

Yamauchi and Kurosaka investigated the presence of stem cells in the bulge area of plucked hair follicles from the scalp [16]. The investigators focused on the presence of glycogen synthase kinase-3 (GSK-3), a protein that, on being inhibited, increases the levels of *β*-catenin directly involved in hair follicle morphogenesis and stem cell differentiation [16]. The presence of GSK-3 in this region was confirmed by looking for its genetic expression by RT-qPCR and by western blotting with a GSK-3 beta-specific antibody, Y174 [16]. Sasahara et al. detected stem cells in the bulge area through the presence of CD34 expression, which is a stem cell biomarker together with other stem cell biomarker genes such as CD200, Sox2, and NANOG [17]. Morphology and expression of keratin family genes in bulge derived follicles (BDKs) before and after differentiation induction with calcium chloride were similar to those of epidermal keratinocytes obtained from skin biopsies (NHEKs) [17]. They also showed that BDKs were more refractory to differentiation than epidermal keratinocytes obtained from skin biopsies [17]. 

### 3.1. Human Follicle-Derived Keratinocytes

Yoshikawa et al. investigated the upregulation of genes that are involved in keratinocyte differentiation, specifically the novel marker gene ID2 [18]. They achieved this by using contact sensitizers in cultured keratinocytes derived from the bulge of plucked haired follicles also known as bulge-derived keratinocytes (BDKs) [18]. Their technique was an efficient and simple method of establishing strains of human BDKs, without the use of invasive skin biopsies [18]. BDKs showed primary responses to sensitizers accompanied by the upregulation of the genes orchestrating keratinocyte differentiation, including the *ID2 *gene and the NRF2-mediated signaling pathway [18]. BDKs were individually established without invasive biopsies, possibly becoming a powerful tool in evaluating donor-to-donor variations to the sensitizers [18].

### 3.2. Stem Cell Reprogramming from Plucked Hair Shaft

Reprogramming of somatic cells into induced pluripotent stem (iPS) cells can be achieved through forced expression of specific transcription factors, notably the combination of Oct4, Sox2, Klf4, and c-Myc (OSKM) [19]. These programmed cells are similar to embryonic stem (ES) cells and are characterized by the unlimited self-renewal potential and the ability to differentiate into any cell type [19]. The technique of generating iPS cells has revolutionized the field investigating the molecular mechanisms of cellular pluripotency and facilitated the generation of patient-specific cells for cell replacement therapy [20]. Ethical and host-rejection issues that are commonly associated with ES cell technology have been reduced, generating great interest and promise for future clinical applications [20]. Reprogramming is slow and inefficient, and the full extent of whether iPS cells can replace ES cells in every aspect is still being debated, and partial reprogramming or “over-reprogramming” poses challenges [21].

Keratinocyte-derived iPS (KiPS) cells can be established from minute amounts of biological sample including plucked hairs. Large numbers of keratinocytes have been successfully cultivated from plucked hair [22]. A single hair plucked from a 30-year-old woman has been used by Aasen et al. to generate keratinocyte induced pluripotent stem cells [23]. An experimental model for investigating the bases of cellular reprogramming and potential advantages of using keratinocytes to generate patient-specific iPS cells is described [23]. Aasen and Belmonte describe a method that uses plucked hair ORS keratinocytes and state that direct plucking of hair will isolate mainly transit-amplifying cells with short-term culture potential [24].

Stem cells derived from plucked hair follicles have been successfully reprogrammed into two very important and relatively inaccessibly tissues, neural cell, and cardiac cells [25]. Reprogramming was achieved by using a single polycistronic excisable lentiviral vector [25]. Novak et al. showed that all the colonies were true iPSCs, had typical characteristics of human embryonic stem cells, and differentiated into all of the three germ layers both *in vitro* and *in vivo *[25]. Consequently, functional cardiac myocytes were successfully derived and characterized from human hair follicle keratinocytes HFKT-iPSCs and exhibited well-coordinated intracellular Ca^2+^ transients and contractions [25].

In another study by Petit and colleagues, keratinocytes that were isolated from hair follicles were found to be an ideal source of patients' cells for reprogramming [26]. They only used a small number of plucked human hair follicles from two healthy donors in order to reprogram keratinocytes into pluripotent stem cells [26]. The group also managed to further differentiate these programmed stem cells to neural progenitors, including forebrain neurons and functional dopaminergic neurons [26].

The review paper by Muller et al. mentions the modelling of human channelopathies plucked hair follicles as a readily available source of iPSCs. Generated iPSCs are considered a useful tool for elucidating pathophysiological mechanisms in various disease states, among which are mentioned diabetes, blood disorders, defined neurological disorders, and genetic liver disease [27]. Linta et al. used human keratinocyte derived induced pluripotent stem cells (hiPSCs) to look at mRNA expression levels of ion channel genes between such cells and their somatic cell source, keratinocytes from plucked human hair [28]. 

## 4. Gene Expression Profiling

Gene expression profiling (GEP) is an important research avenue for understanding how cells and tissues function under normal conditions, characterizing the responses to toxicological or pharmaceutical exposures and elucidating molecular mechanisms associated with aging, disease development, and progression. Several authors have used RT-qPCR to analyze the expression of a limited number of genes in adult plucked human hair follicles [29–31]. Kim et al. highlight the fact that RNA of sufficient quantity and quality to be used in microarray hybridizations could be obtained from a single plucked human hair follicle [32]. The average quantifiable yield of RNA/follicle was 112.5 ng [32]. Ribosomal ratios were lower than normally expected, but investigation indicated that the RNA was intact [32]. The full records of genes expressed in hair follicles of each of the 10 subjects investigated in their study were deposited with the gene expression omnibus (GEO, http://www.ncbi.nlm.nih.gov/geo/) [32].

Ohyama et al. showed that human bulge cell biology could be facilitated by analysis of global gene expression profiles and identification of unique cell-surface markers [33]. Studies of bulge cells have been hampered by the lack of distinctive bulge morphology in human hair follicles [33]. Using navigated laser capture microdissection, the authors determined the distribution of label-retaining cells to define the human anagen bulge [33]. Gene transcripts encoding inhibitors of WNT and activin/bone morphogenic protein signaling were overrepresented in the bulge, while genes responsible for cell proliferation were underrepresented, consistent with the existence of quiescent noncycling KSCs in anagen follicles [33].

Comparative gene expression profiling has been used to distinguish atopic eczema from nonatopic eczema [34]. Human hair follicle-derived keratinocytes (FDKs) were cultured from plucked hairs taken from the two groups [34]. Microarray analysis and quantitative RT-PCR were used to generate gene expression signatures that can distinguish atopic dermatitis from nonatopic controls without skin biopsies [34]. Patient-derived FDKs, individually established without invasive biopsies, may be an ideal cell source to study skin diseases *in vitro* [34].

## 5. Diagnostic and Clinical Applications of Plucked Hair

Plucked hair shafts are becoming a useful diagnostic tool in dermatological conditions. Direct immunofluorescence (DIF) of perilesional skin is the gold standard in the diagnosis of pemphigus [35]. Rao et al. have used the ORS of plucked anagen hair shafts to detect pemphigus specific immunofluorescence pattern and concluded that DIF of plucked hair is a simple, noninvasive test that in future may alleviate the need for skin biopsies in patients with pemphigus [35].

### 5.1. Autologous Epidermal Equivalent

Keratinocytes of the outer root sheath of plucked anagen hair follicles were employed by Tausche et al. to generate fully differentiated autologous epidermal equivalents [36]. They report results from a multicenter, randomized phase II study for EpiDex, a trade mark of a tissue-engineered, fully differentiated autologous epidermal equivalent which was as effective as split-thickness skin autografting in the promotion of healing and complete closure of recalcitrant vascular leg ulcers [36]. Limat et al. used autologous *in vitro* reconstructed epidermal equivalents and showed that, after 2 months, one third of recurrent leg ulcers could be healed [7]. The use of autologous keratinocytes isolated from plucked human scalp hair follicles was shown to offer a number of advantages, including the easy, noninvasive isolation of ORS keratinocytes from plucked anagen hair follicles and their ability to maintain a high proliferative capacity in culture, even when derived from very old donors [7].

### 5.2. Three-Dimensional Skin Equivalents

Three-dimensional skin equivalents (SE) have been used in pharmacological and toxicological research [37] to replace animal experiments [38] and cell monocultures [39]. Furthermore, they are successful tools for grafting of chronic wounds or burned skin and in transplantation medicine. Hoeller et al. developed an improved and rapid method to construct autologous SEs from human plucked hair follicles and fibroblasts [40]. By using anagen phase plucked hair shafts that were chosen by light microscopy and implanting them in dermal equivalents, the process of generating autologous SE was shortened from 30 to 20 days [40].

### 5.3. Surrogate Tissue in Pharmacokinetic/Pharmacodynamic Studies

Plucked hair follicles have joined the list of surrogate tissues for cancer research together with peripheral blood mononuclear cells (PBMCs), platelet-rich plasma, skin biopsies, and oral buccal swaps. Tissue-based approaches to study pharmacodynamic endpoints in early phase oncology clinical trials have widened since the development of targeted drug therapies where the optimal biologic dose would be preferred to the maximally tolerated dose. The definition of optimal dose may be established based on pharmacokinetic end points or, preferably, by demonstrating the desired effect on the target molecule.

 The authors Camidge et al. have described the use of plucked hair follicles and the feasibility in detecting and quantifying cell cycle and DNA repair related factors, such as Ki67, pRb, p27 and phosphorylated p27, pRb, and histone [10]. The effect of antitumor inhibitors of phosphatidylinositol-3-kinase (PI-3-K)/Akt (protein kinase B) signaling in cancer patients has been measured by Williams et al. [41]. Plucked scalp hair follicles were used as surrogate normal tissues to measure the effects of inhibitors of PtdIns-3-kinase and Akt on PtdIns-3-kinase signalling [41]. The study demonstrated that phosphoSer473-Akt staining in the keratinocytes of the external sheath of hair was inhibited by a PtdIns-3-kinase inhibitor within cultured human hair [41]. The results of the study suggest that individual human hairs could provide a minimally invasive way of measuring the effects of PtdIns-3-kinase signaling inhibitors in patients reflecting inhibition of tumor phospho-Akt [41]. 

Plucked hair shafts extracted from the eyebrows as well as peripheral-blood mononuclear cells have been used by Fong et al. as surrogate tissue to test the hypothesis that patients with tumours associated with BRCA1 or BRCA2 mutations would show an objective antitumour response to olaparib, a novel and potent, orally active poly(adenosine diphosphate [ADP]-ribose) polymerase (PARP) inhibitor [42]. In a phase 1 clinical trial, the safety, the adverse-event profile, the dose-limiting toxicity, the maximum tolerated dose, the dose at which PARP is maximally inhibited, and its pharmacokinetic and pharmacodynamic profiles were investigated [42]. Plucked eyebrow hair follicles together with peripheral blood mononuclear cells were used to confirm PARP inhibition in these surrogate samples [42]. Similarly, Ang et al. reviewed the rationale, advantages and disadvantages, and the practical considerations of tissue-based approaches to perform pharmacodynamic studies in early phase oncology clinical trials using case histories of molecular targeting agents such as PI3K, m-TOR, HSP90, HDAC, and PARP inhibitors [43].

## 6. Perspectives

The plucked hair shaft has been used in medical research over the last 60 years. This “mini” organ is becoming an important testing ground in biomedical research. A most exciting role for plucked hair shafts is developing in the field of stem cell reprogramming and the development of autologous epidermal equivalents. The use of plucked hair shaft as surrogate tissue in phase 1 trials for chemotherapeutic drug development as well as its use as a surrogate tissue model in systems biology approaches to medical research will become more important in the immediate future.
